# Supplementary material for: The Evolution and Characterization of the RNA Interference Pathways in Lophotrochozoa
Source: Genome Biol Evol. 2024 May 7;16(5):evae098. doi: 10.1093/gbe/evae098 (PMC11114477; doi:10.1093/gbe/evae098)
Supplement: evae098_Supplementary_Data [file evae098_supplementary_data.zip › Supplementary_materials.zip!/selected_miRNAs/NoGe_selected_miRNAs/mirdeep_pdf_files/NMRB01002279.1_Notospermus_geniculatus_strain_Ushimado_scaffold2279,_whole_genome_shotgun_sequence_37124.pdf]

Provisional ID : NMRB01002279.1_Notospermus_geniculatus_strain_Ushimado_scaffold2279,_whole_genome_shotgun_sequence_37124
Score total : 30550.9
Score for star read(s) : 3.9
Score for read counts : 30542.7
Score for mfe : 1.3
Score for randfold : 0
Score for cons. seed : 3
Total read count : 59920
Mature read count : 59868
Loop read count : 0
Star read count : 52

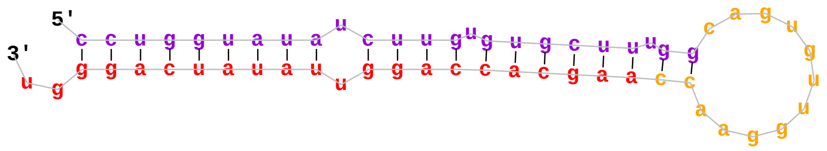

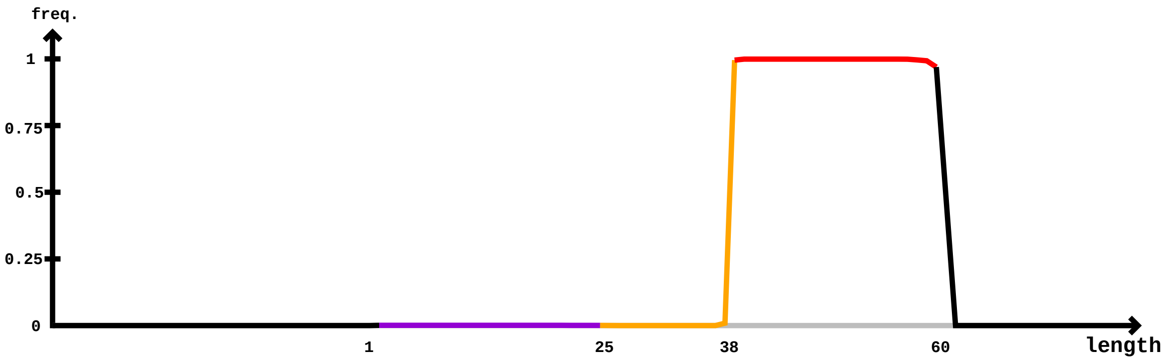

Star Mature

```
5'- cguuauaaucgagcuuaaugccuuacuugaacccugguauaucuugugugcuuuggcaguguuggaaccaagcaccagguuauaucagggugcuugcaucgcauguacaaa  -3'   obs
    cguuauaaucgagcuuaaugccuuacuugaacccugguauaucuugugugcuuuggcaguguuggaaccaagcaccagguuauaucagggugcuugcaucgcauguacaaa         exp
    ........(((((............)))))(((((((((((.((((.((((((.((..........)))))))))))).)))))))))))((.(((...))).)).....  reads  mm  sample
    ................................ccugguauaucuugugugcu...........................................................  5      0   Fm1
    .....................................................................aagcaccagguuauauca........................  2      0   Fm1
    .....................................................................aagcGccagguuauaucag.......................  1      1   Fm1
    .....................................................................aagcaccagguuauaucaU.......................  1      1   Fm1
    .....................................................................aagcaccGgguuauaucag.......................  1      1   Fm1
    .....................................................................aagcaccagguuauaucag.......................  2      0   Fm1
    .....................................................................aagcaccagguuauaucagg......................  5      0   Fm1
    .....................................................................aagcaccagguuauaucaggg.....................  121    0   Fm1
    .....................................................................aagcaccaggAuauaucaggg.....................  1      1   Fm1
    .....................................................................aagcaccagguuauauAaggg.....................  1      1   Fm1
    .....................................................................aagcaccagguuauaucGggg.....................  1      1   Fm1
    .....................................................................aagcaccagguuauaucaggAu....................  2      1   Fm1
    .....................................................................aagcaccagguuGuaucagggu....................  2      1   Fm1
    .....................................................................aagcaccagUuuauaucagggu....................  1      1   Fm1
    .....................................................................Nagcaccagguuauaucagggu....................  2      1   Fm1
    .....................................................................aagcaccUgguuauaucagggu....................  2      1   Fm1
    .....................................................................aagcaccagguuauaucaAggu....................  3      1   Fm1
    .....................................................................aagcaccagguuauaucagUgu....................  2      1   Fm1
    .....................................................................aagcaccagguuauaucGgggu....................  37     1   Fm1
    .....................................................................aagcaccagguuauaucaggUu....................  2      1   Fm1
    .....................................................................aagcaccagguuauaucUgggu....................  37     1   Fm1
    .....................................................................aagcUccagguuauaucagggu....................  2      1   Fm1
    .....................................................................aagcaccagguuauaucagggu....................  9116   0   Fm1
    .....................................................................aagcaccagguuaAaucagggu....................  1      1   Fm1
    .....................................................................aagcaccagguuauaucagggA....................  5      1   Fm1
    .....................................................................aagcaccagguuauauAagggu....................  39     1   Fm1
    .....................................................................aaUcaccagguuauaucagggu....................  1      1   Fm1
    .....................................................................Uagcaccagguuauaucagggu....................  7      1   Fm1
    .....................................................................aagcaccagguuauGucagggu....................  1      1   Fm1
    .....................................................................aNgcaccagguuauaucagggu....................  1      1   Fm1
    .....................................................................aagcaccagguuauaucCgggu....................  27     1   Fm1
    .....................................................................aagcaccaggCuauaucagggu....................  10     1   Fm1
    .....................................................................aagcaNcagguuauaucagggu....................  2      1   Fm1
```

Star Mature

```
cguuauaaucgagcuuaaugccuuacuugaacccugguauaucuugugugcuuuggcaguguuggaaccaagcaccagguuauaucagggugcuugcaucgcauguacaaa
.....................................................................aagcaccagguuauaCcagggu....................   1     1     Fm1
.....................................................................aagUaccagguuauaucagggu....................   2     1     Fm1
.....................................................................aagcacUagguuauaucagggu....................   6     1     Fm1
.....................................................................aagcaccagguuauauGagggu....................   10    1     Fm1
.....................................................................aagcaccagguuauaucaggCu....................   1     1     Fm1
.....................................................................aagcaccagguuauaAcagggu....................   1     1     Fm1
.....................................................................aagcaccagguuauaucagggC....................   115   1     Fm1
.....................................................................aagcaccGgguuauaucagggu....................   59    1     Fm1
.....................................................................Gagcaccagguuauaucagggu....................   4     1     Fm1
.....................................................................aagcaccagguuauaucagAgu....................   1     1     Fm1
.....................................................................aagcaccaggGuauaucagggu....................   7     1     Fm1
.....................................................................aaAcaccagguuauaucagggu....................   3     1     Fm1
.....................................................................aagcaGcagguuauaucagggu....................   1     1     Fm1
.....................................................................aagcaccaggAuauaucagggu....................   13    1     Fm1
.....................................................................aagcaccagguuauauUagggu....................   22    1     Fm1
.....................................................................aagcaccCgguuauaucagggu....................   1     1     Fm1
.....................................................................aGgcaccagguuauaucagggu....................   4     1     Fm1
.....................................................................aagcaccagguuauaucaUggu....................   3     1     Fm1
.....................................................................aagcaccagguuauaucagggG....................   175   1     Fm1
.....................................................................aaCcaccagguuauaucagggu....................   1     1     Fm1
.....................................................................aagcacAagguuauaucagggu....................   1     1     Fm1
.....................................................................aagcaccagguuauUucagggu....................   2     1     Fm1
.....................................................................aagcaccagguuauaucaggguA...................   97    1     Fm1
.....................................................................aagcaccagguuauaucaggguU...................   589   1     Fm1
......................................................................agcaccagguuauaucaggg.....................   2     0     Fm1
......................................................................agcaccagguuauaucaggUu....................   1     1     Fm1
......................................................................agcaccagguuauaucagggu....................   28    0     Fm1
......................................................................agcaccagguAauaucagggu....................   1     1     Fm1

.....................................................................aagcaccagguuauaucagg......................   20    0     Fm2
.....................................................................aagcaccagguuaAaucagg......................   1     1     Fm2
.....................................................................aagcaccagguuauaucaggg.....................   48    0     Fm2
.....................................................................aagcaUcagguuauaucagggu....................   1     1     Fm2
.....................................................................aagcaccaggCuauaucagggu....................   5     1     Fm2
.....................................................................aagcaccagguuauauGagggu....................   4     1     Fm2
.....................................................................aagcaccaggGuauaucagggu....................   2     1     Fm2
.....................................................................aagcaccagguuauaucaggAu....................   1     1     Fm2
.....................................................................aagcacUagguuauaucagggu....................   1     1     Fm2
.....................................................................aagcaccagguuauaucUgggu....................   10    1     Fm2
.....................................................................aagcaccagguuauaucGgggu....................   9     1     Fm2
.....................................................................aagAaccagguuauaucagggu....................   1     1     Fm2
.....................................................................aGgcaccagguuauaucagggu....................   9     1     Fm2
.....................................................................aaUcaccagguuauaucagggu....................   3     1     Fm2
.....................................................................aagcaccagguuauaucagggG....................   47    1     Fm2
.....................................................................aagcaccagguuauauAagggu....................   8     1     Fm2
.....................................................................aagcaccagguuauaucagAgu....................   1     1     Fm2
.....................................................................aagcaccagguuaAaucagggu....................   2     1     Fm2
.....................................................................aagcaccagguuauaucaggUu....................   1     1     Fm2
.....................................................................Uagcaccagguuauaucagggu....................   1     1     Fm2
.....................................................................aagcaccagguuauaucagggA....................   1     1     Fm2
.....................................................................aagcaccagguuauaucCgggu....................   7     1     Fm2
.....................................................................aagcaccagguuauaucagggC....................   27    1     Fm2
.....................................................................Gagcaccagguuauaucagggu....................   1     1     Fm2
.....................................................................aagcaccaggAuauaucagggu....................   5     1     Fm2
.....................................................................aagcaccagguuauauUagggu....................   6     1     Fm2
.....................................................................aagcaccagguuGuaucagggu....................   2     1     Fm2
.....................................................................aagcaccGgguuauaucagggu....................   10    1     Fm2
.....................................................................aagcaccagguuauaucagggu....................   2526  0     Fm2
.....................................................................aagcaccagguuauaucagUgu....................   2     1     Fm2
.....................................................................aagcaccagguuauaucaggguU...................   126   1     Fm2
.....................................................................aagcaccagguuauaucaggguA...................   37    1     Fm2
.....................................................................aagcaccagguuauaucagggug...................   1     0     Fm2

.....................................................................aagcaccagguuauauca........................   8     0     Mm1
.....................................................................aagcaccagguuauaucag.......................   1     0     Mm1
.....................................................................aagcaccagguuauaucagg......................   4     0     Mm1
.....................................................................aagcaccGgguuauaucaggg.....................   1     1     Mm1
.....................................................................aagcaccagguuauauUaggg.....................   1     1     Mm1
.....................................................................aagcaccagguuauaucGggg.....................   2     1     Mm1
.....................................................................aagcaccagguuauaucaggg.....................   140   0     Mm1
```

```
                                Star                                  Mature
cguuauaaucgagcuuaaugccuuacuugaacccugguauaucuugugugcuuuggcaguguuggaaccaagcaccagguuauaucagggugcuugcaucgcauguacaaa
.....................................................................aagcaccagguuauaucCgggu....................   6      1    Mm1
.....................................................................aagcaccaggGuauaucagggu....................   2      1    Mm1
.....................................................................aagcaccagguuauGucagggu....................   1      1    Mm1
.....................................................................Nagcaccagguuauaucagggu....................   1      1    Mm1
.....................................................................aagAaccagguuauaucagggu....................   1      1    Mm1
.....................................................................aagcaccagguuauaucGgggu....................   2      1    Mm1
.....................................................................aagcaccagguuauaucagggu....................   1242   0    Mm1
.....................................................................Gagcaccagguuauaucagggu....................   1      1    Mm1
.....................................................................aagcacUagguuauaucagggu....................   1      1    Mm1
.....................................................................aagcaccagguuauaucUgggu....................   4      1    Mm1
.....................................................................aagcGccagguuauaucagggu....................   1      1    Mm1
.....................................................................aaUcaccagguuauaucagggu....................   2      1    Mm1
.....................................................................aagcaccagguuauauAagggu....................   5      1    Mm1
.....................................................................aagcaccagguuauauGagggu....................   3      1    Mm1
.....................................................................aagcaccagguuauaucagggC....................   74     1    Mm1
.....................................................................aagcaccaggAuauaucagggu....................   4      1    Mm1
.....................................................................aagcaccagguuauauUagggu....................   4      1    Mm1
.....................................................................aagcaccagguuauaucagggG....................   1      1    Mm1
.....................................................................Uagcaccagguuauaucagggu....................   1      1    Mm1
.....................................................................aagcaccagguuauaucaggguU...................   13     1    Mm1
.....................................................................aagcaccagguuauaucaggguA...................   35     1    Mm1
......................................................................agcaccagguuauaucagggu....................   5      0    Mm1

.............cuuaaugccuuacuugaac...............................................................................   1      0    Mm2
................................ccugguauaucuugugugcu...........................................................   2      0    Mm2
................................ccugguauaucuugugugcuuu.........................................................   2      0    Mm2
................................ccugguauaucuugugugcuuug........................................................   1      0    Mm2
................................ccugguauaucuugugugcuuugg.......................................................   14     0    Mm2
........................................Uucuugugugcuuuggca.....................................................   1      1    Mm2
...................................................................Ucaagcaccagguuauaucagggu....................   2      1    Mm2
....................................................................caagcaccagguuauaucaggg.....................   6      0    Mm2
....................................................................caagcaccagguuauauAaggg.....................   1      1    Mm2
....................................................................caagcaccagAuuauaucagggu....................   1      1    Mm2
....................................................................caagcacAagguuauaucagggu....................   1      1    Mm2
....................................................................caagcaccagguuauaucagggu....................   6      0    Mm2
....................................................................caagcaccagguuauaucagggC....................   1      1    Mm2
.....................................................................aagcaccagguuauauca........................   2      0    Mm2
.....................................................................aagcaccagguuauaucGg.......................   1      1    Mm2
.....................................................................aagcaccagguuauaucaA.......................   1      1    Mm2
.....................................................................aagcaccagguuauaucag.......................   7      0    Mm2
.....................................................................aagcaccagguuauaucUgg......................   1      1    Mm2
.....................................................................aagcaccGgguuauaucagg......................   1      1    Mm2
.....................................................................aagcaccagguuauaucagg......................   14     0    Mm2
.....................................................................aagcaccagguuauaucGggg.....................   2      1    Mm2
.....................................................................aagcaccagguuauaucCggg.....................   1      1    Mm2
.....................................................................aagcaccagguuauauAaggg.....................   1      1    Mm2
.....................................................................aagcaccagguuGuaucaggg.....................   1      1    Mm2
.....................................................................aagcaccagguuauaucaggg.....................   348    0    Mm2
.....................................................................aagcaccGgguuauaucaggg.....................   4      1    Mm2
.....................................................................aagcaccagguuauaCcaggg.....................   1      1    Mm2
.....................................................................aagcaccaggAuauaucaggg.....................   1      1    Mm2
.....................................................................aagcaccagguuauauUaggg.....................   3      1    Mm2
.....................................................................aagcaccagguuauaucUggg.....................   2      1    Mm2
.....................................................................aaAcaccagguuauaucaggg.....................   2      1    Mm2
.....................................................................aagcaccagguuauauGaggg.....................   1      1    Mm2
.....................................................................aagcaccagguuauaucaggU.....................   1      1    Mm2
.....................................................................aagcaccagguuauaucaggA.....................   1      1    Mm2
.....................................................................aaUcaccagguuauaucagggu....................   4      1    Mm2
.....................................................................aagcaccagguuaAaucagggu....................   2      1    Mm2
.....................................................................Gagcaccagguuauaucagggu....................   2      1    Mm2
.....................................................................aagcaccagguuaCaucagggu....................   2      1    Mm2
.....................................................................aagcaccagguuauaucagggC....................   302    1    Mm2
.....................................................................aagcaccagguuauGucagggu....................   3      1    Mm2
.....................................................................aagcacUagguuauaucagggu....................   12     1    Mm2
.....................................................................aGgcaccagguuauaucagggu....................   7      1    Mm2
.....................................................................aagcaccaggAuauaucagggu....................   43     1    Mm2
.....................................................................aagcaccagguuauauAagggu....................   82     1    Mm2
.....................................................................aUgcaccagguuauaucagggu....................   1      1    Mm2
.....................................................................aagcaccagguuauaucagCgu....................   1      1    Mm2
.....................................................................aagcCccagguuauaucagggu....................   2      1    Mm2
```

Star Mature

```
cguuauaaucgagcuuaaugccuuacuugaacccugguauaucuugugugcuuuggcaguguuggaaccaagcaccagguuauaucagggugcuugcaucgcauguacaaa
.....................................................................aagcaccagguuauauGagggu....................   23      1   Mm2
.....................................................................aagcaccagguuauaucagggG....................   330     1   Mm2
.....................................................................aagcaccagguuauaucaUggu....................   1       1   Mm2
.....................................................................aagcaccaUguuauaucagggu....................   2       1   Mm2
.....................................................................aagcaccagguUuaucagggu....................   4       1   Mm2
.....................................................................aagcaccagguuauaucaAggu....................   1       1   Mm2
.....................................................................aagcacNagguuauaucagggu....................   2       1   Mm2
.....................................................................aagcaccagguuGuaucagggu....................   1       1   Mm2
.....................................................................aagcaNcagguuauaucagggu....................   4       1   Mm2
.....................................................................aagcaccaggCuauaucagggu....................   23      1   Mm2
.....................................................................aagcaccagguuauaucaggCu....................   6       1   Mm2
.....................................................................aagcaGcagguuauaucagggu....................   1       1   Mm2
.....................................................................aagcaccagguGauaucagggu....................   2       1   Mm2
.....................................................................aagcaccagguuauUucagggu....................   3       1   Mm2
.....................................................................Uagcaccagguuauaucagggu....................   16      1   Mm2
.....................................................................aagcaccagguuaGaucagggu....................   1       1   Mm2
.....................................................................aagcaUcagguuauaucagggu....................   2       1   Mm2
.....................................................................aCgcaccagguuauaucagggu....................   2       1   Mm2
.....................................................................aagcaccagUuuauaucagggu....................   1       1   Mm2
.....................................................................aagGaccagguuauaucagggu....................   1       1   Mm2
.....................................................................aagcaccagguuauaAcagggu....................   1       1   Mm2
.....................................................................aagcaccagAuuauaucagggu....................   2       1   Mm2
.....................................................................aagcaccagguuauaucGgggu....................   78      1   Mm2
.....................................................................aagcaccGgguuauaucagggu....................   76      1   Mm2
.....................................................................aagcaccUgguuauaucagggu....................   4       1   Mm2
.....................................................................aagcaccCgguuauaucagggu....................   1       1   Mm2
.....................................................................aagcaccagguuauaCcagggu....................   3       1   Mm2
.....................................................................aagcaccagguuauaucagggA....................   10      1   Mm2
.....................................................................aagcaccagguuauaucagggu....................   20898   0   Mm2
.....................................................................aagcaccagCuuauaucagggu....................   1       1   Mm2
.....................................................................aagcaccagguuauauUagggu....................   52      1   Mm2
.....................................................................aagcacAagguuauaucagggu....................   1       1   Mm2
.....................................................................aaCcaccagguuauaucagggu....................   1       1   Mm2
.....................................................................aagcacGagguuauaucagggu....................   1       1   Mm2
.....................................................................aagcaccagguCauaucagggu....................   4       1   Mm2
.....................................................................aaAcaccagguuauaucagggu....................   6       1   Mm2
.....................................................................aagcaccagguuauaucagAgu....................   2       1   Mm2
.....................................................................aagcaccagguuauaucCgggu....................   80      1   Mm2
.....................................................................aagAaccagguuauaucagggu....................   5       1   Mm2
.....................................................................aagcaccagguuauaucUgggu....................   75      1   Mm2
.....................................................................aagcGccagguuauaucagggu....................   2       1   Mm2
.....................................................................aagcaccagguuauaucagUgu....................   1       1   Mm2
.....................................................................aagcaccagguuauaucaggAu....................   1       1   Mm2
.....................................................................Nagcaccagguuauaucagggu....................   18      1   Mm2
.....................................................................aagcaccaggGuauaucagggu....................   22      1   Mm2
.....................................................................aagcaccagguuauaucaggguU...................   569     1   Mm2
.....................................................................aagcaccagguuauaucagggug...................   1       0   Mm2
.....................................................................aagcaccagguuauaucaggguA...................   178     1   Mm2
......................................................................agcaccagguuauaucaggg.....................   1       0   Mm2
......................................................................agcaccagguuauaucagggu....................   67      0   Mm2
......................................................................agcaccagguuauaucagggC....................   1       1   Mm2
......................................................................agcaccaCguuauaucagggu....................   1       1   Mm2
......................................................................agcaccagguuauaucagggG....................   2       1   Mm2
......................................................................agcaccaggAuauaucagggu....................   1       1   Mm2
......................................................................agcaccagguuauaucCgggu....................   1       1   Mm2
......................................................................agcaccagguuauaucaggguU...................   1       1   Mm2
......................................................................agcaccagguuauaucaggguC...................   1       1   Mm2
.......................................................................gcaccagguuauaucagggu....................   1       0   Mm2

................................ccugguauaucuugugugcu...........................................................   6       0   Fm3
................................ccugguauaucuugugugcuuug........................................................   1       0   Fm3
................................ccugguauaucuugugugcuuuggA......................................................   3       1   Fm3
....................................................................caagcaccagguuauauca........................   1       0   Fm3
....................................................................caagcaccagguuauaucag.......................   3       0   Fm3
....................................................................caagcaccagguuauauAagg......................   1       1   Fm3
....................................................................caagcaccagguuauaucagg......................   21      0   Fm3
....................................................................caagcaccagguuauauGaggg.....................   1       1   Fm3
....................................................................caagcaccagguuauaucaggg.....................   34      0   Fm3
....................................................................caagcaccagguuauaGcagggu....................   1       1   Fm3
....................................................................caagcaccagUuuauaucagggu....................   1       1   Fm3
```

	Star	Mature	
cguuauaaucgagcuuaaugccuuacuugaacccugguauaucuugugugcuuuggcaguguuggaaccaagcaccagguuauaucagggugcuugcaucgcauguacaaa			
....caagcaccagguuauaucagggu....................	423	0	Fm3
....caagcacUagguuauaucagggu....................	1	1	Fm3
....caagcaccagguuauaAcagggu....................	1	1	Fm3
....caagcaccagguuauauUagggu....................	3	1	Fm3
....caagcaccagguuauaucagggC....................	14	1	Fm3
....caagcaccagguuauaucaUggu....................	1	1	Fm3
....Uaagcaccagguuauaucagggu....................	1	1	Fm3
....caagcaccagguuauauGagggu....................	2	1	Fm3
....caagcaccagCuuauaucagggu....................	2	1	Fm3
....caagcaccagguuauaCcagggu....................	1	1	Fm3
....caagcaccagAuuauaucagggu....................	1	1	Fm3
....caagcaccagguuauauAagggu....................	1	1	Fm3
....caagcaccagguuauaucaggguU...................	6	1	Fm3
....aagcaccagguuauauAa........................	1	1	Fm3
....aagcaccagguuauauca........................	4	0	Fm3
....aagcaccagguuauaucag.......................	136	0	Fm3
....aagcaccagguuauauAag.......................	2	1	Fm3
....aagcaccaggAuauaucag.......................	1	1	Fm3
....aagcaccagguuauaucagU......................	3	1	Fm3
....aagcaccagguuaCaucagg......................	1	1	Fm3
....aagcaccagguuauaucagg......................	80	0	Fm3
....aagcaccagguuauauGagg......................	1	1	Fm3
....aagcaccagguuauauAagg......................	1	1	Fm3
....aagcaccagguuauaucaggU.....................	2	1	Fm3
....Uagcaccagguuauaucaggg.....................	2	1	Fm3
....aagcaccagguuauauGaggg.....................	1	1	Fm3
....aagcaccGgguuauaucaggg.....................	1	1	Fm3
....aagcaccagguuauaucCggg.....................	1	1	Fm3
....aagcaccagguuauaCcaggg.....................	1	1	Fm3
....aagcaccagguuauauUaggg.....................	3	1	Fm3
....Nagcaccagguuauaucaggg.....................	1	1	Fm3
....aagcaccaggCuauaucaggg.....................	2	1	Fm3
....aagcaccaggAuauaucaggg.....................	2	1	Fm3
....aagcaccagguuauaucaggg.....................	455	0	Fm3
....aagcaccagguuauaucUggg.....................	1	1	Fm3
....aagcaccagCuuauaucaggg.....................	1	1	Fm3
....aagcaccagguuauCucagggu....................	1	1	Fm3
....aagcaccaggAuauaucagggu....................	26	1	Fm3
....aaAcaccagguuauaucagggu....................	2	1	Fm3
....aagcaccUgguuauaucagggu....................	4	1	Fm3
....Uagcaccagguuauaucagggu....................	9	1	Fm3
....aagcaccGgguuauaucagggu....................	9	1	Fm3
....aagcaccagguuauauUagggu....................	32	1	Fm3
....aagcaccagguuauaucaggAu....................	2	1	Fm3
....aagcaccagguuauGucagggu....................	5	1	Fm3
....aagcaccagguuauaucaAggu....................	1	1	Fm3
....aagcaccagAuuauaucagggu....................	3	1	Fm3
....aagcaccagguuauaucCgggu....................	48	1	Fm3
....aagcGccagguuauaucagggu....................	3	1	Fm3
....aagcaccagguuauaucGgggu....................	43	1	Fm3
....aagcaccagguuCuaucagggu....................	1	1	Fm3
....aagcacAagguuauaucagggu....................	5	1	Fm3
....aagcaccagguuauaucaggUu....................	1	1	Fm3
....aagcaccaAguuauaucagggu....................	1	1	Fm3
....Nagcaccagguuauaucagggu....................	12	1	Fm3
....aagcaccagguuauaucaUggu....................	6	1	Fm3
....aagcCccagguuauaucagggu....................	1	1	Fm3
....aagcaccagguuauaucagUgu....................	5	1	Fm3
....aagcaccagguuauaucUgggu....................	43	1	Fm3
....aagcaccagguuauaucagAgu....................	1	1	Fm3
....aagcaccagguuauauGagggu....................	21	1	Fm3
....aagAaccagguuauaucagggu....................	4	1	Fm3
....aagcaUcagguuauaucagggu....................	8	1	Fm3
....aCgcaccagguuauaucagggu....................	2	1	Fm3
....aagcaccagguuauauAagggu....................	74	1	Fm3
....Gagcaccagguuauaucagggu....................	2	1	Fm3
....aagcaccagguuauaucagggu....................	13074	0	Fm3
....aGgcaccagguuauaucagggu....................	4	1	Fm3
....aagcaccaggCuauaucagggu....................	13	1	Fm3
....aagcaccagguuUuaucagggu....................	1	1	Fm3

```
                               Star                                   Mature
cguuauaaucgagcuuaaugccuuacuugaacccugguauaucuugugugcuuuggcaguguuggaaccaagcaccagguuauaucagggugcuugcaucgcauguacaaa
.....................................................................aagcaccagguuauUucagggu....................   1      1   Fm3
.....................................................................aagcaccagguuGuaucagggu....................   1      1   Fm3
.....................................................................aagcacUagguuauaucagggu....................   6      1   Fm3
.....................................................................aagcaccagguuauaAcagggu....................   1      1   Fm3
.....................................................................aagcaccaggGuauaucagggu....................   18     1   Fm3
.....................................................................aaUcaccagguuauaucagggu....................   1      1   Fm3
.....................................................................aagcaccagguuaCaucagggu....................   2      1   Fm3
.....................................................................aaCcaccagguuauaucagggu....................   1      1   Fm3
.....................................................................aagcaccagguuauaucagggC....................   101    1   Fm3
.....................................................................aUgcaccagguuauaucagggu....................   1      1   Fm3
.....................................................................aagcaccagguuauaucagCgu....................   1      1   Fm3
.....................................................................aagcaccaUguuauaucagggu....................   3      1   Fm3
.....................................................................aagcacNagguuauaucagggu....................   1      1   Fm3
.....................................................................aagcaccagguuaAaucagggu....................   1      1   Fm3
.....................................................................aagcaccagguuauaucagggA....................   8      1   Fm3
.....................................................................aagcaccagguuauaucaggguU...................   382    1   Fm3
.....................................................................aagcaccagguuauaucaggguA...................   85     1   Fm3
......................................................................agcaccagguuauaucaggg.....................   1      0   Fm3
......................................................................agcaccagguuaAaucaggg.....................   1      1   Fm3
......................................................................agcaccagguuauaucagggC....................   2      1   Fm3
......................................................................agcaccaggGuauaucagggu....................   1      1   Fm3
......................................................................agcaccagguuauaucaggUu....................   1      1   Fm3
......................................................................agcaccagguAauaucagggu....................   1      1   Fm3
......................................................................agcaccagguuauaucagggu....................   72     0   Fm3
......................................................................agcaccaggCuauaucagggu....................   1      1   Fm3
......................................................................agcaccaggAuauaucagggu....................   1      1   Fm3
......................................................................agcaccCgguuauaucagggu....................   1      1   Fm3
......................................................................agcaccagguuauaAcagggu....................   1      1   Fm3
......................................................................agcaccagguuauaGcagggu....................   1      1   Fm3
......................................................................agcaccagguuauaucaggguU...................   6      1   Fm3

................................ccugguauaucuugugugcu...........................................................   1      0   Mm3
................................ccugguauaucuugugugcuu..........................................................   1      0   Mm3
................................ccugguauaucuugugugcuuug........................................................   2      0   Mm3
................................ccugguauaucuugugugcuuugg.......................................................   13     0   Mm3
....................................................................caagcaccagguuauaucaggg.....................   1      0   Mm3
....................................................................caagcaccagguuauaucagggu....................   1      0   Mm3
.....................................................................aagcaccagguuauaucag.......................   3      0   Mm3
.....................................................................aagcaccagguuauaucagg......................   41     0   Mm3
.....................................................................aagcaccagguuauaucCgg......................   1      1   Mm3
.....................................................................aagcGccagguuauaucaggg.....................   1      1   Mm3
.....................................................................aagcaccagguuauaucaggA.....................   1      1   Mm3
.....................................................................aagcaccGgguuauaucaggg.....................   1      1   Mm3
.....................................................................aagcaccaggCuauaucaggg.....................   1      1   Mm3
.....................................................................aagcaccagguuauaucaggg.....................   205    0   Mm3
.....................................................................aCgcaccagguuauaucagggu....................   1      1   Mm3
.....................................................................aagcUccagguuauaucagggu....................   1      1   Mm3
.....................................................................aagcaccagguuauaucagggG....................   45     1   Mm3
.....................................................................aagcaccagguuauaucagggC....................   144    1   Mm3
.....................................................................aagcaccagguuauauAagggu....................   17     1   Mm3
.....................................................................aagcaccagguuauaucagUgu....................   3      1   Mm3
.....................................................................aagcaccagguuauauUagggu....................   15     1   Mm3
.....................................................................aagcaccagguuauaucaggUu....................   2      1   Mm3
.....................................................................aagcaccagguuauaucagggu....................   5061   0   Mm3
.....................................................................aagcaccagUuuauaucagggu....................   2      1   Mm3
.....................................................................aagcaccagguuauaucCgggu....................   23     1   Mm3
.....................................................................aaAcaccagguuauaucagggu....................   1      1   Mm3
.....................................................................aagGaccagguuauaucagggu....................   1      1   Mm3
.....................................................................aagcaccagguuCuaucagggu....................   1      1   Mm3
.....................................................................aagcaccagguuauaucagggA....................   5      1   Mm3
.....................................................................Uagcaccagguuauaucagggu....................   3      1   Mm3
.....................................................................aagcaccagguuauaucaggCu....................   1      1   Mm3
.....................................................................aagcaccagguuauaucUgggu....................   18     1   Mm3
.....................................................................aagAaccagguuauaucagggu....................   3      1   Mm3
.....................................................................aagcaccagguuauaAcagggu....................   1      1   Mm3
.....................................................................aagcaccagguuauauGagggu....................   4      1   Mm3
.....................................................................aagcacNagguuauaucagggu....................   1      1   Mm3
.....................................................................aagcaccagguuauaucGgggu....................   14     1   Mm3
.....................................................................aagcaccaggCuauaucagggu....................   7      1   Mm3
.....................................................................aagcaccaUguuauaucagggu....................   1      1   Mm3
```

```
                               Star                                   Mature
cguuauaaucgagcuuaaugccuuacuugaacccugguauaucuugugugcuuuggcaguguuggaaccaagcaccagguuauaucagggugcuugcaucgcauguacaaa
.....................................................................Nagcaccagguuauaucagggu....................  1    1  Mm3
.....................................................................aagcaccGgguuauaucagggu....................  16   1  Mm3
.....................................................................Gagcaccagguuauaucagggu....................  1    1  Mm3
.....................................................................aagcGccagguuauaucagggu....................  1    1  Mm3
.....................................................................aagcaccaggGuauaucagggu....................  3    1  Mm3
.....................................................................aagcaccUgguuauaucagggu....................  2    1  Mm3
.....................................................................aagcaUcagguuauaucagggu....................  2    1  Mm3
.....................................................................aagcacUagguuauaucagggu....................  2    1  Mm3
.....................................................................aagcaccaggAuauaucagggu....................  11   1  Mm3
.....................................................................aagcaccagguCauaucagggu....................  1    1  Mm3
.....................................................................aagcaccagguuaAaucagggu....................  1    1  Mm3
.....................................................................aagcaccagguuGuaucagggu....................  3    1  Mm3
.....................................................................aagcacGagguuauaucagggu....................  1    1  Mm3
.....................................................................aUgcaccagguuauaucagggu....................  1    1  Mm3
.....................................................................aGgcaccagguuauaucagggu....................  4    1  Mm3
.....................................................................aagcaccagguuauGucagggu....................  2    1  Mm3
.....................................................................aaUcaccagguuauaucagggu....................  3    1  Mm3
.....................................................................aagcaccagguuauaucaAggu....................  2    1  Mm3
.....................................................................aagcaccagguuauUucagggu....................  1    1  Mm3
.....................................................................aagcaccagAuuauaucagggu....................  1    1  Mm3
.....................................................................aagcaccagguuauaucaggguA...................  54   1  Mm3
.....................................................................aagcaccagguuauaucaggguU...................  157  1  Mm3
.....................................................................aagcaccagguuauaucaggguUc..................  1    1  Mm3
......................................................................agcaccagguuauaucagggu....................  12   0  Mm3
......................................................................agcaccaggGuauaucagggu....................  1    1  Mm3
......................................................................agcaccagguuauaucagggA....................  1    1  Mm3
......................................................................agcaccagguuauaucagggC....................  4    1  Mm3
```
